# Predicting the Impact of Temperature Change on the Future Distribution of Maize Stem Borers and Their Natural Enemies along East African Mountain Gradients Using Phenology Models

**DOI:** 10.1371/journal.pone.0130427

**Published:** 2015-06-15

**Authors:** Sizah Mwalusepo, Henri E. Z. Tonnang, Estomih S. Massawe, Gerphas O. Okuku, Nancy Khadioli, Tino Johansson, Paul-André Calatayud, Bruno Pierre Le Ru

**Affiliations:** 1 CHIESA Project, *icipe*—African Insect Science for Food and Health, Nairobi, Kenya; 2 Department of Mathematics, University of Dar es Salaam, Dar es Salaam, Tanzania; 3 Department of General studies, Dar es Salaam Institute of Technology, Dar es Salaam, Tanzania; 4 NSBB Project, *icipe*—African Insect Science for Food and Health, Nairobi, Kenya; 5 IRD/CNRS UMR IRD 247 EGCE, Laboratoire Evolution Génomes Comportement et Ecologie, CNRS, Gif sur Yvette Cedex, France; 6 Université Paris-Sud 11, Orsay, France; Pennsylvania State University, UNITED STATES

## Abstract

Lepidopteran stem borers are among the most important pests of maize in East Africa. The objective of the present study was to predict the impact of temperature change on the distribution and abundance of the crambid *Chilo partellus*, the noctuid *Busseola fusca*, and their larval parasitoids *Cotesia flavipes* and *Cotesia sesamiae* at local scale along Kilimanjaro and Taita Hills gradients in Tanzania and Kenya, respectively. Temperature-dependent phenology models of pests and parasitoids were used in a geographic information system for mapping. The three risk indices namely establishment, generation, and activity indices were computed using current temperature data record from local weather stations and future (i.e., 2055) climatic condition based on downscaled climate change data from the AFRICLIM database. The calculations were carried out using index interpolator, a sub-module of the Insect Life Cycle Modeling (ILCYM) software. Thin plate algorithm was used for interpolation of the indices. Our study confirmed that temperature was a key factor explaining the distribution of stem borers and their natural enemies but other climatic factors and factors related to the top-down regulation of pests by parasitoids (host-parasitoid synchrony) also played a role. Results based on temperature only indicated a worsening of stem borer impact on maize production along the two East African mountain gradients studied. This was attributed to three main changes occurring simultaneously: (1) range expansion of the lowland species *C*. *partellus* in areas above 1200 m.a.s.l.; (2) increase of the number of pest generations across all altitudes, thus by 2055 damage by both pests will increase in the most productive maize zones of both transects; (3) disruption of the geographical distribution of pests and their larval parasitoids will cause an improvement of biological control at altitude below 1200 m.a.s.l. and a deterioration above 1200 m.a.s.l. The predicted increase in pest activity will significantly increase maize yield losses in all agro-ecological zones across both transects but to a much greater extent in lower areas.

## Introduction

The average global surface temperature has increased by about 0.6°C during the past century [1,2,3,4,5], mainly as a result of human activities [2,3,4,6]. Continental and sea temperatures are expected to continue to rise regardless of human interventions for at least the next decades [2,3,4]. It is anticipated that the impact of climate change will be greater in the tropical regions of the world. In addition, various socio-economic studies, and present demographic and policy trends suggest that developing countries will be more at risk because of their low capacity to adapt [3]. This will exacerbate the already serious challenges to food security, livelihoods and economic prosperity [7,8].

Climatic predictions show that Eastern Afromontane Biodiversity Hotspot (EABH) regions will be particularly affected by climatic changes [9,10,11,12,13]. The goods and services provided by these ecosystems are under significant threat [7,14,15], mostly due to land use changes exacerbated through high population. The EABH have important ecosystem service values arising from the water towers it provides for the low lying areas, food production from major crops like maize, cabbages, and plantation crops like coffee and avocado, recreation and eco-tourism, and nutrient recycling [10].

In EABH, agricultural productivity of crops grown for human consumption is at risk due to the incidence of arthropod pests, which reduce crop production by up to 18% [16,17]. Because insects are cold-blooded, they cannot regulate their own temperatures, and hence, temperature is considered among the major abiotic factor affecting insect development, reproduction, survival and thereby their interaction with the environment (plant and natural enemies), and geographic distribution [18,19,20,21,22,23].

The responses of insects to temperature has been given much more attention in temperate than in tropical regions [24,25,26]. In temperate regions, warmer winter and decreasing frequencies of temperature extremes will enhance reproduction capacity and changes in distribution are expected for a variety of pest species [18] whereas abundance of some insect species vulnerable to high temperatures may decrease as a result of climate change [27,28,29]. Nonetheless, tropical regions are more predisposed to insect pest problems and outbreaks because of favorable climatic conditions for pest reproduction and host availability throughout the year [26]. One of the best approaches to predict the consequences of climate change and especially temperature on insect distribution is the use of phenological models [30]. Different models have been used to estimate the relationship between temperature and insect development [26,31,32,33,34]. Nonlinear models simulate the variability in insect development time within a population based on detailed laboratory assessment of the insect’s life history and, thus, can provide good results on future pest activities [35,36]. Furthermore, they allow prediction of pest population dynamics in different agro-ecological zones in response to climate change by the use of spatial models [26,37].

Maize is the most important staple food in East African countries [38,39,40,41]. Maize production is constrained by biotic (stem borers, gray leaf sport, maize streak virus) and abiotic (drought, low soil fertility) factors. In East Africa, the most important insect pests associated with maize are lepidopteran stem borers, among them the crambid *Chilo partellus* (Swinhoe) and the noctuid *Busseola fusca* (Fuller) are the most detrimental. Yield losses are currently estimated between 12% to 40% of the total production depending on agro-climatic zone, maize variety, cropping system, and soil fertility level [39,40,42,43].

Distribution and relative importance of *C*. *partellus* and *B*. *fusca* varies among different regions and agroecological zone. *C*. *partellus* invaded Africa from Asia sometime before 1930 and was first reported from Malawi [44]. Currently, it has spread to nearly all countries in East and Southern Africa, and became the most serious pest of cereals in the hot lowlands and the mid altitudes [45,46,47,48]. *B*. *fusca* is indigenous to Africa and the predominant pest in the cooler highlands. However, its distribution varies between African regions; while in East and Southern Africa it is a pest in the cooler zones above 600 m, in Central Africa it the predominant pest from sea level to over 2000 m, and in West Africa it is primarily a pest of sorghum in the dry savannahs [48,49].

Both pests are attacked by natural enemies, among them the larval parasitoids *Cotesia sesamiae* (Cameron) and *Cotesia flavipes* Cameron (Hymenoptera: Braconidae) which attack *B*. *fusca and C*. *partellus*, respectively [42]. Whereas, *C*. *sesamiae* is indigenous to Africa, *C*. *flavipes*, a native of Asia, was released in Kenya in 1993 in a classical biological control program against *C*. *partellus* [50]. The natural enemies are reported to cause 32–55% decrease in stem borer densities in various cereal crops [42,47].

Temperature-driven phenology models for stem borers, which include a set of functions describing temperature-dependency to determine the pests`life history, have been used to forecast *C*. *partellus* development in Africa [51,52]. While pest models and risk mapping tools have been shown to be useful for predictions on global and regional scales, there is a general lack of information for predictions of maize stem borers at a smaller geographical scale. EABH regions, characterized by a hilly relief with steep inclines, are particularly affected by this situation due to restricted computational facilities, inadequate human resources, problems associated with the insufficiency of regional climate data and reliable downscaling options [2,7,11]. As maize is the most important staple crop in EABH, more in-depth research is needed about the likely effects that climate change will have on its predominant insect pests and their main parasitoids.

The hypothesis of the present study is that the distribution and abundance of maize stem borers will shift towards higher elevations and interactions with their natural enemies is expected to change as a consequence of climate change along East African mountain gradients. Therefore, the objective of this study was to predict the impact of climate changes on the distribution and abundance of *B*. *fusca*, *C*. *partellus*, *C*. *flavipes* and *C*. *sesamiae* along two EABH gradients through phenology modelling.

## Material and Methods

### Study sites

**Fig 1:**
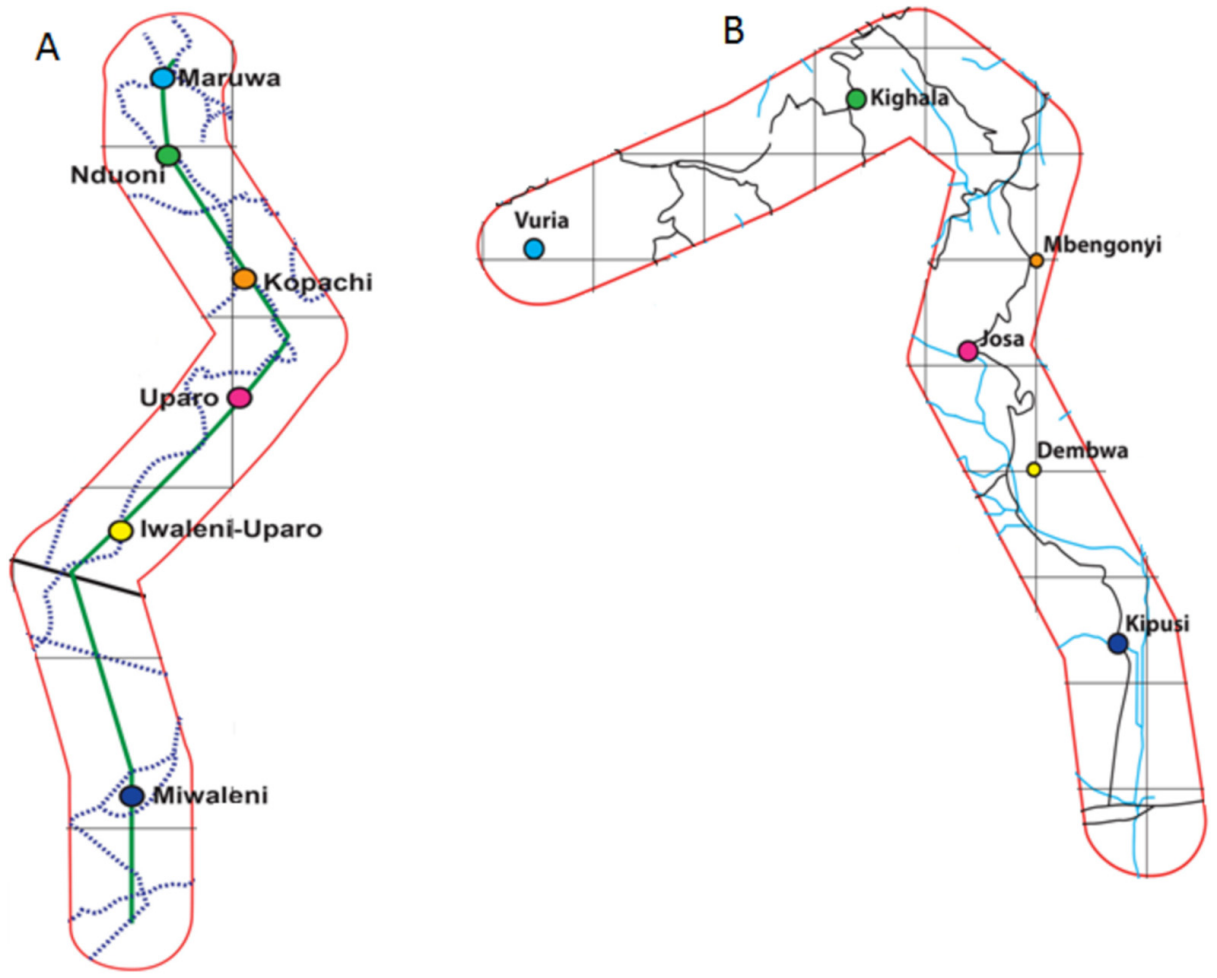
The study areas: (A) Mount Kilimanjaro transect, (B) Taita hills transect. The circles indicate the locations where the data loggers for the climate data collection have been installed along the altitudinal gradients.

The study areas are localized in the Eastern Afromontane Biodiversity Hostspot (EABH) in Kenya and Tanzania. In Kenya, the target area is situated in the Taita Hills in the Coast Province to the south-east, at an elevation ranging from 700 to 2228 m.a.s.l., between latitude 3°25´ and longitude 38°20´ (Fig 1). Mean annual rainfall ranges from 500 mm to over 1500 mm and mean annual temperature from 16.5 to 23.5°C in, respectively, the low altitudes and upper mountain zone. The area is characterized by a bimodal rainfall distribution, with a long rainy season occurring from March to May/June and a short one from September/October to December. In Tanzania, the target area is situated in the Pangani river basin in the north east with focus on the small catchment areas on the south-eastern slope of Mount Kilimanjaro, at 700–1800 m.a.s.l., between latitude 3°4´ and longitude 37°4´ (Fig 1). Mean annual temperature ranges from 18 to 23.6°C and mean annual rainfall between 1000mm to 1300mm. It experiences two distinct rainy seasons: a long season from March to May and a short one between October and December. Permissions to carry research by CHIESA project were granted by Kenya Forest Service under reference RESEA/1/KFS/5 for Taita Hills and by the Regional Administrative Secretary Kilimanjaro under reference FA/191/228/01/61 for Kilimanjaro mountains.

### Stem borer data

Field surveys were carried out in maize fields along the two-altitudinal gradients for two years in 2012 and 2013. No specific permissions were required for these maize fields; however permissions to conduct research were asked to the small-scale farmers owners of the maize plots. The field studies did not involve endangered or protected species. A total of 6 localities along each altitudinal transect were selected on the basis of the annual mean temperature, whereby each locality differed from the closest one by 1°C. In addition these localities belong to some of the maize agro-ecological zones defined by Hassan *et al*., [53]. The lowland tropical and dry mid altitude zones situated mainly below 1300 m.a.s.l belong to low potential zones, whereas the moist transitional and highland tropics located above 1300 m.a.s.l belong to high potential zones for maize. Ten maize plots were selected in each locality. Twelve infested plants were sampled every 5–6 weeks in each plot and dissected. All stem borers recovered were sorted according to their developmental stage and species name if possible, counted and placed individually in a glass vial containing artificial diet developed by Onyango and Ochieng’-Odero [54]. Each vial was labeled according to the larval stage, species name, location, plot number and date. The larvae were taken to the laboratory and kept until parasitism emergence or pupae formation. Parasitoids cocoons recovered were recorded and the parasitoids identified. Pupae were kept until adult emergence for confirmation of species identity.

### Climate data

The temperature data required for carrying out spatial simulation under current climatic conditions were obtained from local weather stations. At each study site across each transect, 24 automatic onsets HOBO data loggers were installed, within a minimum distance of 600m from each others, to keep track of daily minimum and maximum temperatures, and monthly mean minimum and maximum temperatures (°C). The following localities were considered: Kipusi, Dembwa, Josa, Mbengonyi, Kighala and Vuria in the Taita Hills, and Miwaleni, Uparo Iwaleni, Uparo, Kopachi, Nduoni and Marua in the Mount Kilimanjaro transect. At each locality, the geographical coordinates (longitude and latitude) and altitudes were recorded using a Global Positioning System (GPS).

For simulations under future climatic conditions, downscaled data of the Representative Concentration Pathways Scenarios, Fifth Assessment Report (RCPs-AR5) [3] under future climate change scenarios were used. The RCPs provide spatially resolved data sets of land use change and sector-based emissions of air pollutants, and it specifies annual greenhouse gas concentrations and anthropogenic emissions up to 2100 [3]. The downscaling of the data was obtained from regional climate models (RCMs). RCMs better capture local climate feedbacks, especially in biologically rich and highly populous mountain and coastal regions. These grids were bias-corrected and change-factor downscaled to 30” (1km) spatial resolution, using the WorldClim grids as baselines. The data are well documented in Platts *et al*., [55], and are freely accessible at http://www.york.ac.uk/environment/research/kite/resources/. For studying the effect of future climate conditions on stem borers and their natural enemies, the year 2055 was selected because of the time horizon of 30 years, which is more realistic than 2080 and beyond.

### The phenology models

The temperature-driven phenology models for *C*. *partellus*, *B*. *fusca*, *C*. *flavipes* and *C*. *sesamiae* were developed based on laboratory data at constant temperatures, and they were validated with life table statistics collected at fluctuating temperatures under field conditions [51,52,56]. Daily minimum and maximum temperatures obtained during these experiments were used in stochastic simulations [51,52]. A good agreement was observed between simulated and experimental results, e.g. for *B*. *fusca*, the simulated development times were 8.86, 67.39 and 19.91 days when the observed development times were 8.92, 68.74 and 19.33 days for eggs, larvae and pupae respectively; similarly for *C*. *partellus*, the simulated development times were 8.30, 46.23 and 13.81 days when the observed development times were 8.22, 48.36 and 14.86 days for eggs, larvae and pupae, respectively [51,52]. These results show that the phenology models developed by Khadioli et al. [51,52], can be applied to predict the insect population abundance and demographic parameters in other agro-ecological zones, what justified their application in the current study in both transects. The phenology models consisted of a set of functions that described temperature-dependent development of the three immature life stages (egg, larva and pupae). The Logit function was used to describe the variation of development time on eggs, larvae and pupae, whereas the complementary ClogLog model was used for female and male pupae [51,52,56,57]. The Logan model [31,51,55] was used to describe the development rate of the stem borer species and their natural enemies. The effect of temperature on the mortality of the immature life stages was described by a second order exponential polynomial function [51,52,55,57,58]. A three-parameter Stinner model [51,55,59] was applied to determine the relationship between longevity of female adults and temperature for *B*. *fusca*, *C*. *partellus* and *C*. *sesamiae*. Hilbert & Logan model [60] was used to describe the effect of temperature on senescence rate for *C*. *flavipes* and Gamma and polynomial functions were chosen to describe effect of temperature on the reproduction.

### Spatial Analysis

Simulations in ILCYM are based on daily minimum and maximum temperatures [26]. In selected geographical coordinates, minimum and maximum temperature are inferred in the phenology model through a cosine function, which was then applied for direct estimate of the following life table parameters: generation time, net reproduction rate, intrinsic rate of increase, finite rate of increase, and doubling time [26,58] From the expression of individual life table parameter, three risk indices namely establishment index (ERI), this index identifies the geographical areas in which the pest insect may survive; generation index (GI), this index is an estimate of the mean number of generations that a given insect may produce within a given year; and activity index (AI), indicates the decimal power of the estimated population increase within a given year [37] were derived for assessing the potential distribution and abundance of the species. This approach implemented the index interpolator in a sub-module of the Insect Life Cycle Modeling (ILCYM) software [57], and is freely accessible at https://research.cip.cgiar.org/confluence/display/ilcym/Home. Using an index interpolator module, the following steps were considered: the digital elevation model (DEM), defined as co-variables, was inputted into ILCYM; DEM was obtained from the Shuttle Radar Topography Mission (SRTM). Phenology model and temperature data from each station for a year were also inputted into the tool. The inbuilt thin plate algorithm was selected for the interpolation of the indices on the surface of DEM. The indices were generated as ASCII files format and were transferred to ArcGIS software version 10.1 for enhancing the visualization. Furthermore, to understand the effect of future climate, the difference between the current (2013) and future (2055) climate condition were obtained by using ILCYM’s inbuilt raster calculator. The corresponding values of absolute generation index changes were extracted using point sampling tool. The generation index changes estimated were plotted against altitude to visualize the change along altitudinal gradients.

The variations of finite rate of increase within the year due to seasonal weather fluctuations were analyzed in detail through plotting the simulated finite rate of increase against calendar days of the year. Two locations per transect were selected to represent the local weather conditions as follows: Miwale (low altitude) and Marua (high altitude) in the Mount Kilimanjaro transect, and Kipusi (low altitude) and Vuria (high altitude) in the Taita hills. The choice of these locations was based on the fact that C. *partellus* is the dominant species at low altitudes, whereas *B*. *fusca* is the dominant species at high altitudes [48].

### Predicting maize yield losses

Based on reports by Hassan et al., [53], De Groote [61], Ong’amo *et al*., [48] a relationship was established between estimated activity indices of the two stem borer species and estimated average maize yield losses associated with stem borer infestations under current climatic conditions in Kenya. Each transect was split into four agro-climatic zones according to altitude as defined by Hassan et al. [53]. From the bottom to the top they included the lowland tropics below 1000 m.a.s.l., a dry mid altitude between 1000–1300 m.a.s.l., a moist transitional zone between 1300–1600 m.a.s.l., and the highland tropics above 1600 m.a.s.l. Using the geographical coordinates of these zones, the corresponding values for activity indices for current and future climatic conditions were extracted as ASCII files. Since the activity index was continuous in the study area, an average activity index for current and future climatic conditions was calculated for each agro-climatic zone where maize is grown. The relationship between estimated yield losses and activity indices for current climate conditions was estimated by a linear equation:

$$Y=\beta_{0}+\beta_{1}A$$ (1)

where *Y* is the estimated yield loss (%) under current climatic conditions, *A* is the activity index representing the potential damage, and *β*_0_ and *β*_1_ are intercept and slope of the equation, respectively. Using the parameter values for *β*_0_ and *β*_1_ and activity index values for the current climate, the predicted yield losses were estimated for current climatic conditions. With the assumption that future yield losses will follow the same relationship as current ones, the linear equation was used to estimate yield losses in study areas under future climatic conditions. Thus, the values for *A* in the above equation were replaced with activity index values for the year 2055 as defined below:

$$Y_{1}=\beta_{0}+\beta_{1}A_{1}$$ (2)

where *Y*_1_ is the estimated yield loss (%) during 2055, and *A*_1_ is the activity index representing the potential damage under future climatic conditions.

## Results

### Change in species distribution

**Fig 2:**
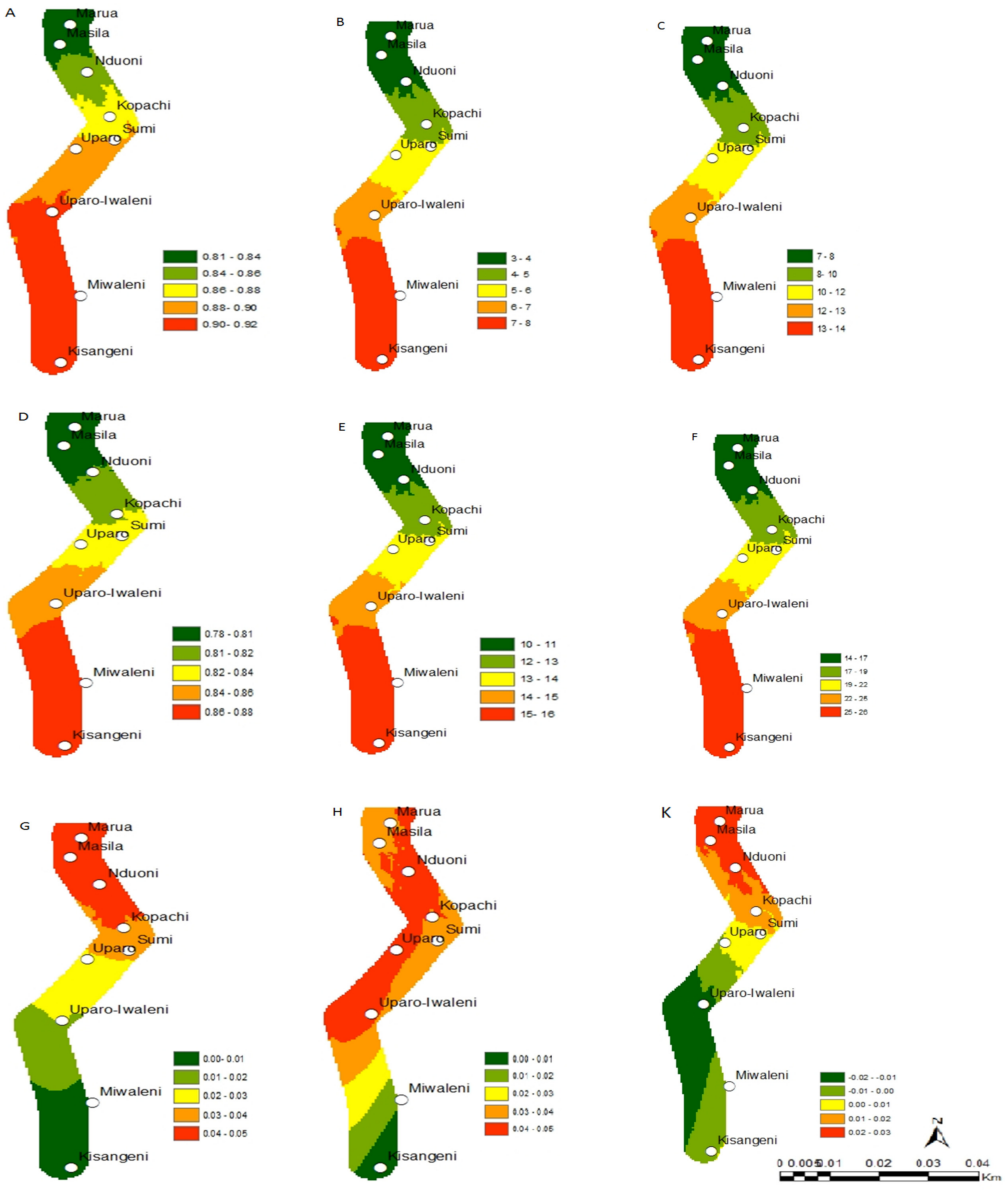
Change in the establishment, abundance and activity indices of *C*. *partellus* and *C*. *flavipes* along the Mount Kilimanjaro transect; *C*. *partellus* current distribution, (A) (ERI), (B) (GI), (C) (AI); *C*. *flavipes* current distribution (D) (ERI), (E) (GI), (F) (AI); absolute establishment index change between 2013 and 2055, (G) *C*. *partellus*, (H) *C*. *flavipes*; (K) *C*. *partellus* and *C*. *flavipes* synchrony under future climate for the establishment index between 2013 and 2055. ERI = Establishment Index, GI = Generation Index, AI = Activity Index.

**Fig 3:**
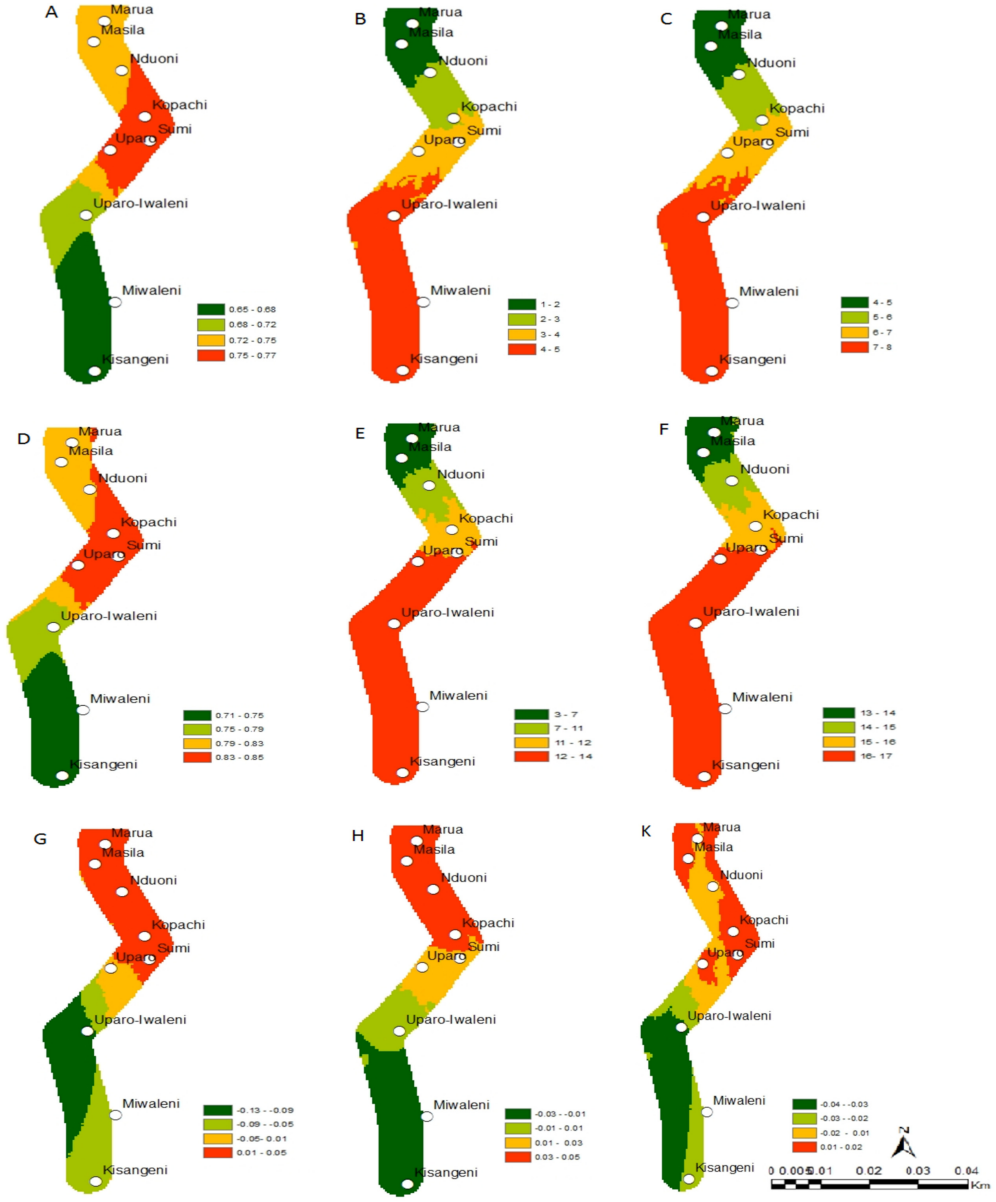
Change in the establishment, abundance and activity indices of *B*. *fusca* and *C*. *sesamiae* along the Mount Kilimanjaro transect; *B*. *fusca* current distribution, (A) ERI, (B) GI, (C) AI; *C*. *sesamiae* current distribution (D) ERI, (E) GI, (F) AI; absolute establishment index change between 2013 and 2055, (G) *B*. *fusca*, (H) *C*. *sesamiae*; (K) *B*. *fusca* and *C*. *sesamiae* synchrony under future climate for the establishment index between 2013 and 2055. ERI = Establishment Index, GI = Generation Index, AI = Activity Index.

**Fig 4:**
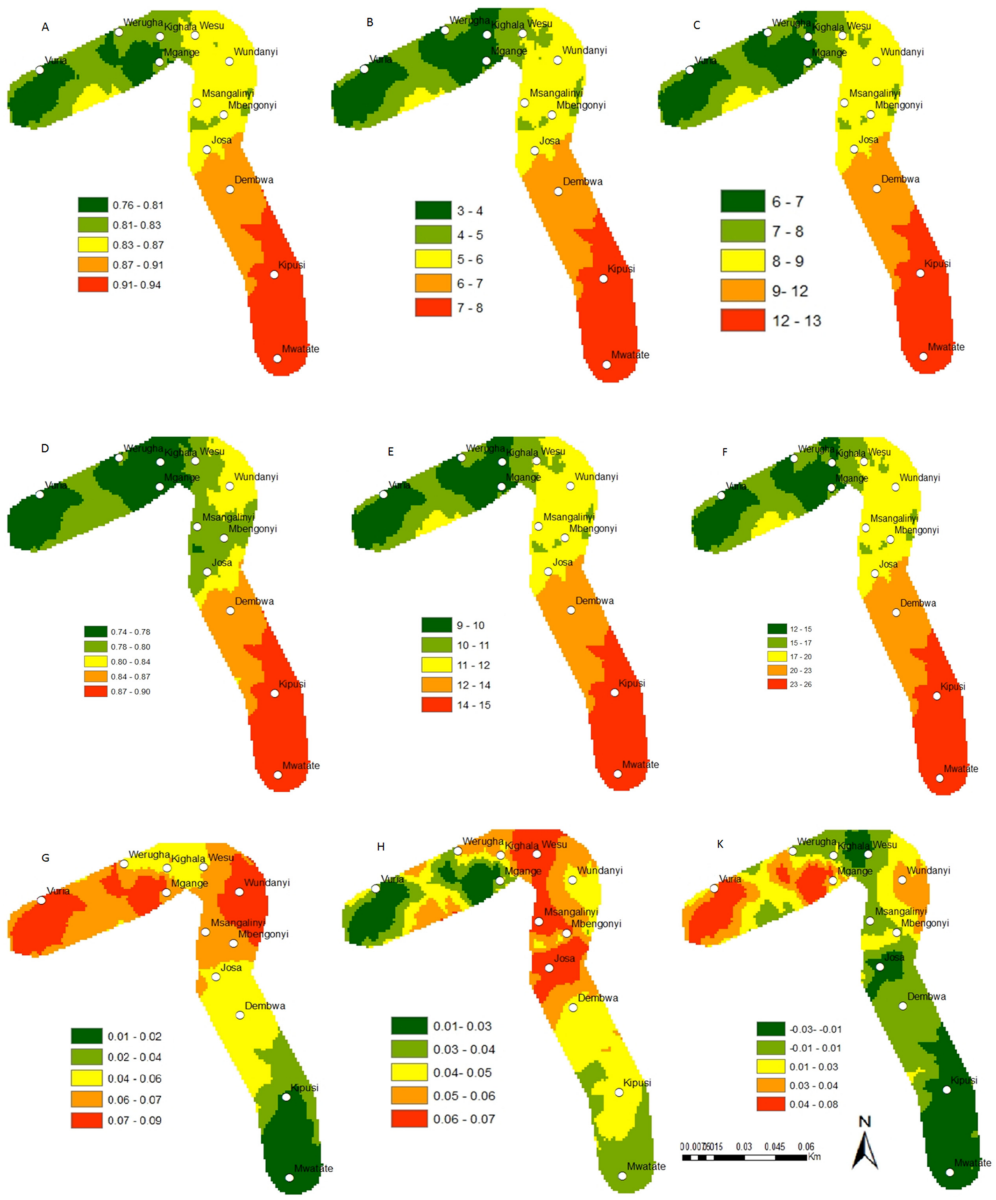
Change in the establishment, abundance and activity indices of *C*. *partellus* and *C*. *flavipes* study along the Taita hills transect; *C*. *partellus* current distribution, (A) (ERI), (B) (GI), (C) (AI); *C*. *flavipes* current distribution (D) (ERI), (E) (GI), (F) (AI); absolute establishment index change between 2013 and 2055, (G) *C*. *partellus*, (H) *C*. *flavipes*; (K) *C*. *partellus* and *C*. *flavipes* synchrony under future climate for the establishment index between 2013 and 2055. ERI = Establishment Index, GI = Generation Index, AI = Activity Index.

**Fig 5:**
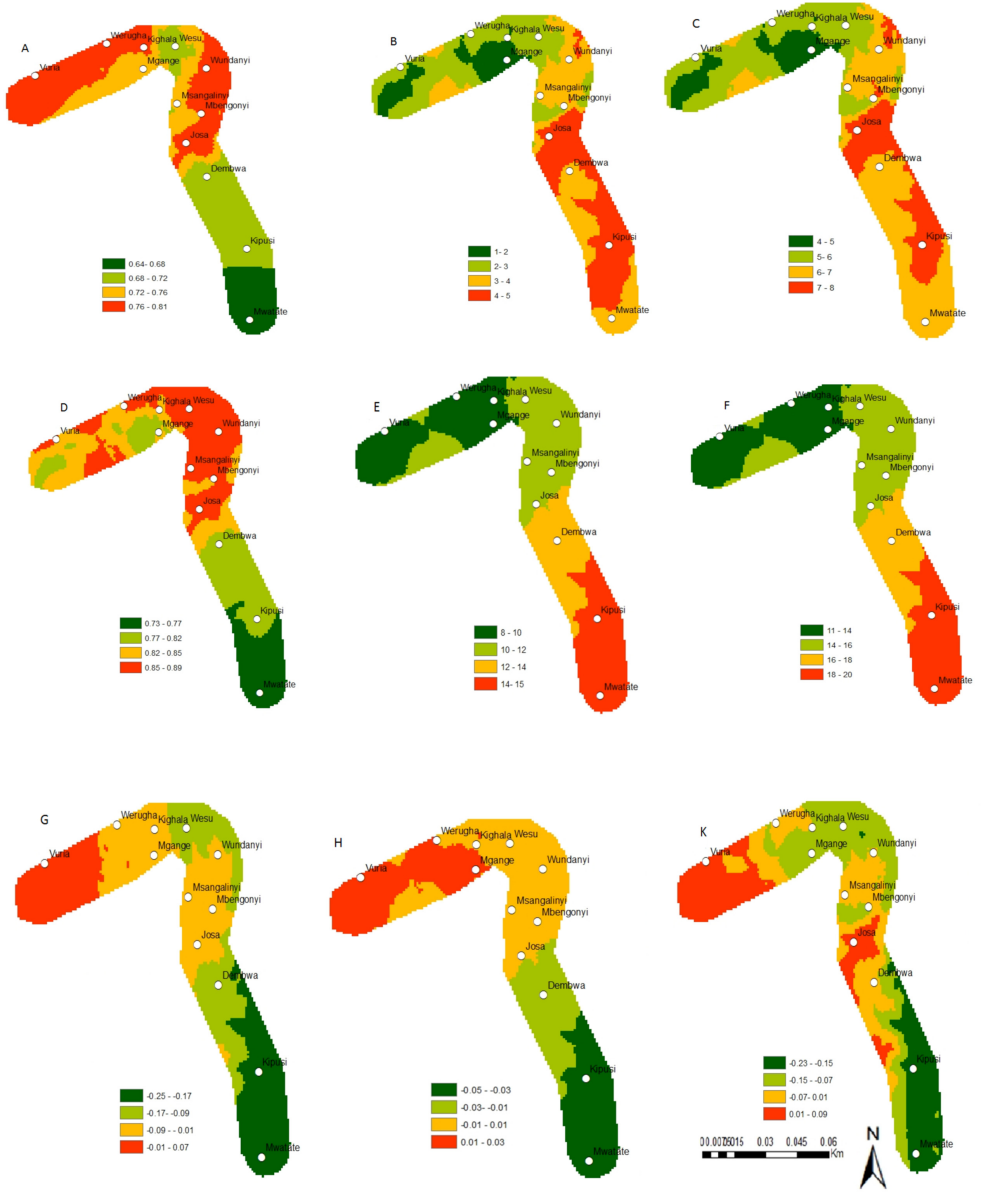
Changes in the establishment, abundance and activity indices of *B*. *fusca* and *C*. *sesamiae* study along the Taita hills transect; *B*. *fusca* current distribution, (A) ERI, (B) GI, (C) AI; *C*. *sesamiae* current distribution (D) ERI, (E) GI, (F) AI; absolute establishment index change between 2013 and 2055, (G) *B*. *fusca*, (H) *C*. *sesamiae*; (K) *B*. *fusca* and *C*. *sesamiae* synchrony under future climate for the establishment index between 2013 and 2055. ERI = Establishment Index, GI = Generation Index, AI = Activity Index.

Under the current climate scenario of the year 2013, the establishment risk index (ERI) ranged from 0.81 to 0.92, 0.65 to 0.77, 0.78 to 0.88 and 0.71 to 0.85 for *C*. *partellus*, *B*. *fusca*, *C*. *flavipes* and *C*. *sesamiae*, respectively in the Mount Kilimanjaro region (Figs 2A,3A,2D and 3D), and from 0.76 to 0.94, 0.64 to 0.81, 0.74 to 0.90 and 0.73 to 0.89 for *C*. *partellus*, *B*. *fusca*, *C*. *flavipes* and *C*. *sesamiae*, respectively in the Taita Hills (Figs 4A, 5A, 4D and 5D). This reflects well the current distribution of *C*. *partellus* with higher values at low altitudes and lower values at high altitudes, and *B*. *fusca* with higher values at high altitudes and lower values at low altitudes. Above 1100 m.a.s.l the likelihood of establishment of *C*. *partellus* is lower but it does occur. Presently, *C*. *partellus* is already distributed below 1600 m.a.s.l and presents a severe risk of establishment (ERI>0.81) in the higher altitudes of the gradient. Under the year 2055 temperature scenario, the boundaries of the four species are indicated to shift to higher altitude with an absolute change in establishment index of up to 0.05 at the top of the gradient in the Mount Kilimanjaro region (Figs 2G, 2H, 3G, and 3H), and up to 0.09, 0.07, 0.07 and 0.03 at the top of the gradient in the Taita Hills for *C*. *partellus*, *B*. *fusca*, C. flavipes and *C*. *sesamiae*, respectively (Figs 4G, 4H, 5G and 5H).

### Change in species abundance

**Fig 6:**
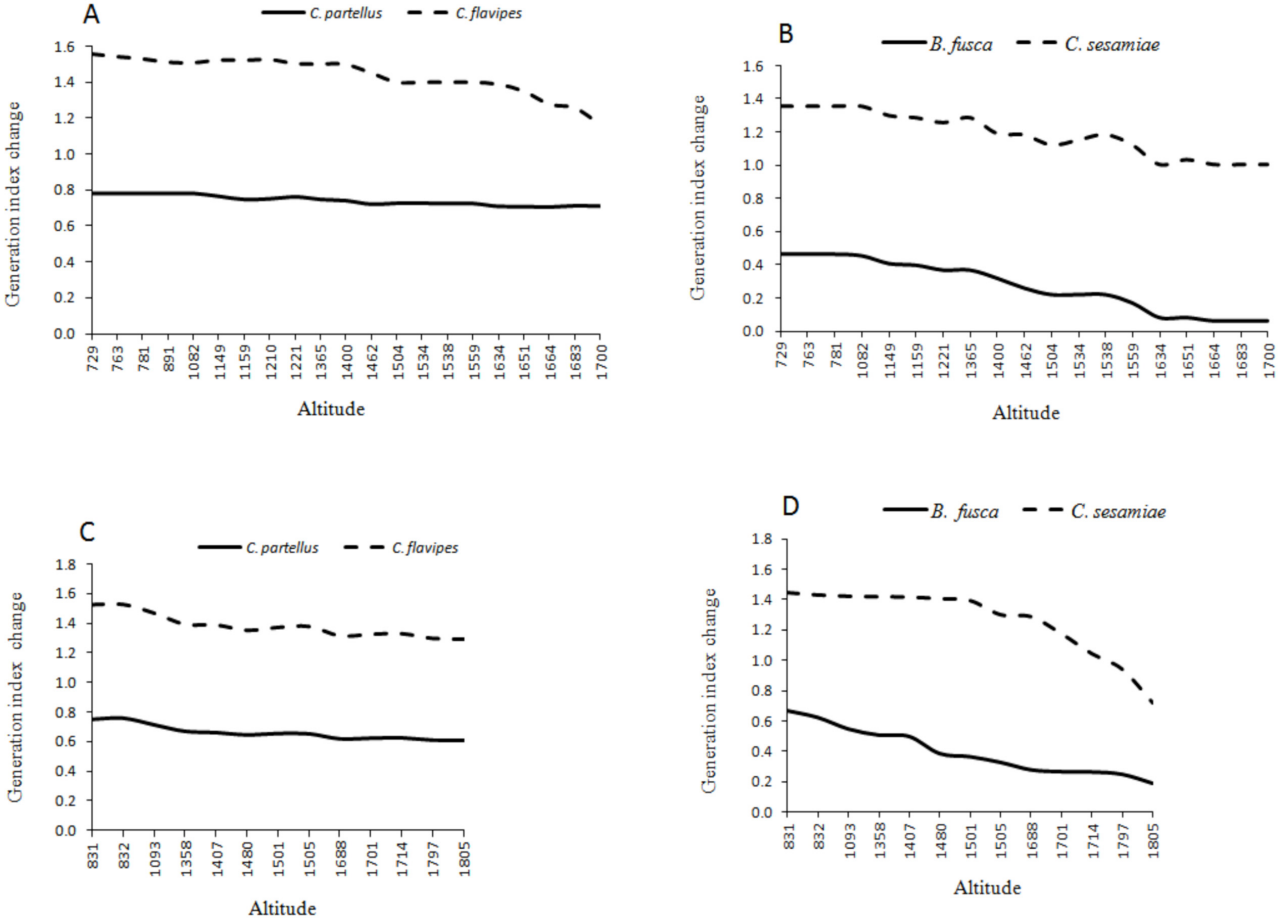
Absolute generation index changes between 2013 and 2055; Mount Kilimanjaro transect. (A) *C*. *partellus* and *C*. *flavipes;* (B) *B*. *fusca* and *C*. *sesamiae*; Taita hills transect, (C) *C*. *partellus* and *C*. *flavipes*, (D) *B*. *fusca* and *C*. *sesamiae*.

Under the current climate of the year 2013, the generation index (GI) (abundance) of *C*. *partellus* fluctuates between 3 in areas around 1400–1600 m.a.s.l and 8 at the bottom of both transects (Figs 2B and 4B). For *C*. *flavipes*, GI ranges from 14–16 in low altitude areas around 700–900 m.a.s.l, and 9–11, respectively at the top of both transect (Figs 2E and 4E). For *B*. *fusca*, GI ranges from 4–5 in low altitude areas around 700–900 m.a.s.l, and 1–2, respectively at the top of both transect (Figs 3B and 5B). *C*. *sesamiae* GI fluctuates between 12–14 in low altitude and 3–7 at the high altitude in the Kilimanjaro transect (Fig 3E); it ranges between 14–15 and 8–10 in the Taita Hills transect at the bottom and the top, respectively (Fig 5E). Under the tear 2055 scenario, the generation index is expected to increase by 0.7–0.8, 0.1–0.5,1.2–1.6, and 1.0–1.4 per year in Mount Kilimanjaro for *C*. *partellus*, *B*. *fusca*, *C*. *flavipes* and *C*. *sesamiae*, respectively (Fig 6A and 6B), and by 0.6–0.7, 0.2–0.7, 1.2–1.5 and 0.7–1.4 per year in the Taita Hills, respectively (Fig 6C and 6D).

The activity index (AI), which is strongly correlated with GI, indicates population growth throughout the year for *C*. *partellus*, varying from 7 to 14 at the bottom and the top of the Kilimanjaro region, respectively (Fig 2C), and, from 6 to 13 at the bottom and the top of the Taita Hills, respectively (Fig 4C). For *B*. *fusca*, AI varies from 4 to 8 at the bottom and the top of both transects (Figs 3C and 5C). AI varies from 23–26 at the bottom to 12–14 at the top on both transects (Figs 2F and 4F) for *C*. *flavipes*; for *C*. *sesamiae* it varies from 16–17 at the bottom to 13–14 at the top of the Kilimanjaro transect (Fig 3F), and between 18–20 and 11–14 at the bottom and the top of the Taita Hills, respectively (Fig 5F).

### Change in species finite rate of increase

**Fig 7:**
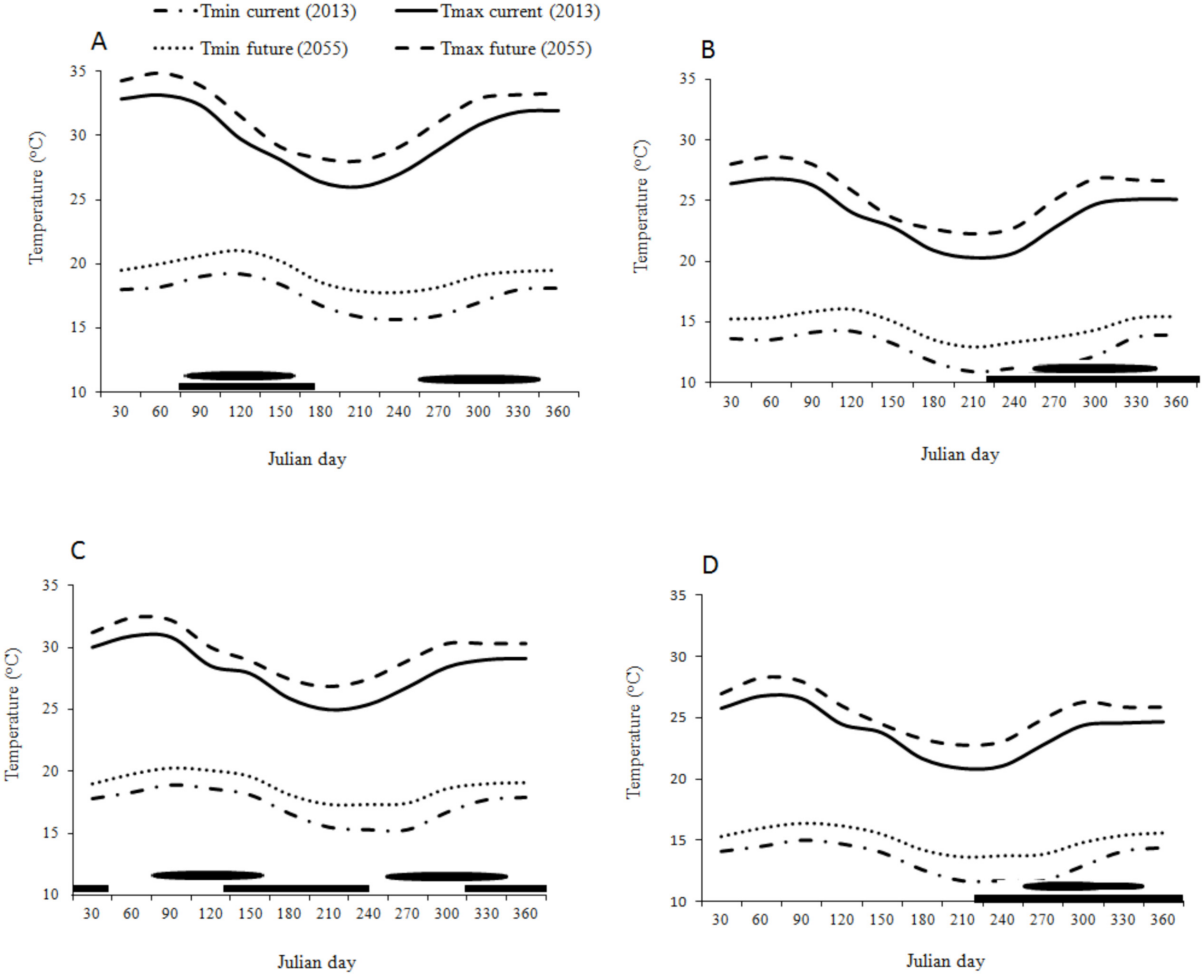
Minimum and maximum temperatures curves for current (2013) and future (2055); (A) Miwaleni (764 m.a.s.l); (B) Marua (1683 m.a.s.l) along Mount Kilimanjaro transect; (C) Kipusi (832 m.a.s.l); (D) Vuria (1800 m.a.s.l) along Taita hills transect. Bars above the x-axis indicate the maize-cropping season and oval indicates the starting of rainy season.

**Fig 8:**
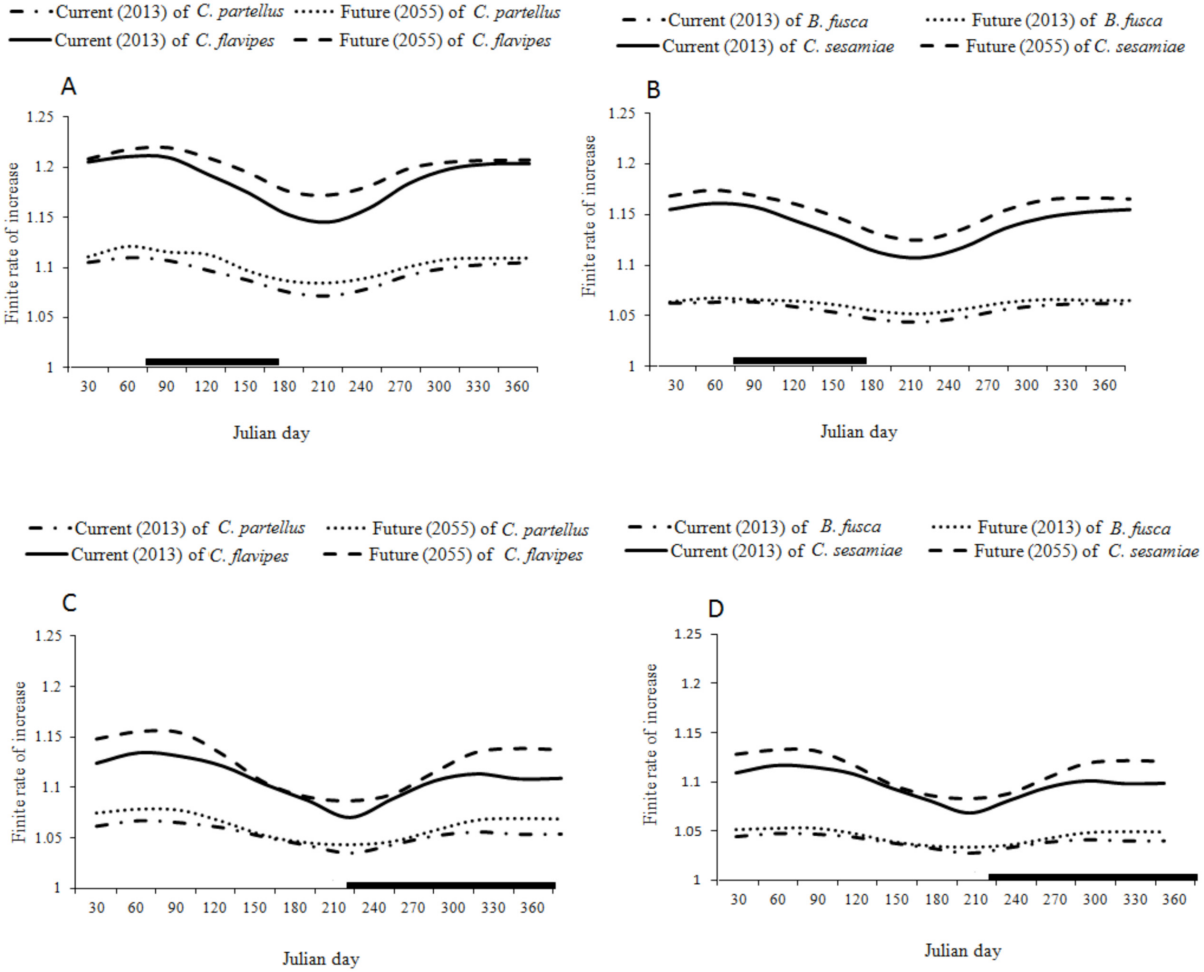
Finite rate of increase for current (2013) and future (2055) throughout the year, at two local weather stations along Mount Kilimanjaro transects; (A) *C*. *partellus* and *C*. *flavipes;* (B) *B*. *fusca* and *C*. *sesamiae* at Miwaleni (764 m.a.s.l); (C) *C*. *partellus* and *C*. *flavipes*; (D) *B*. *fusca* and *C*. *sesamiae* at Marua (1683 m.a.s.l).

**Fig 9:**
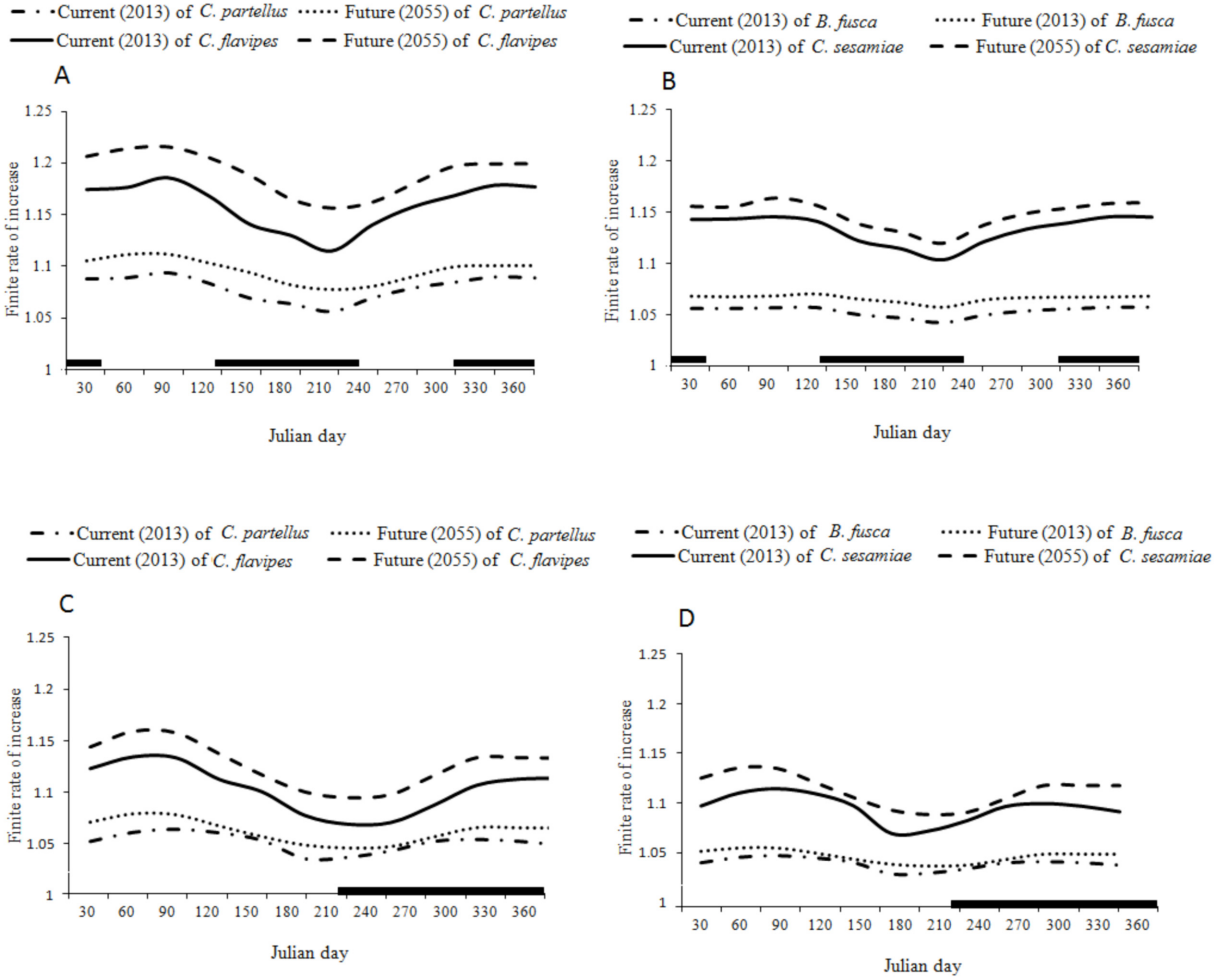
Finite rate of increase for current (2013) and future (2055) throughout the year, at two local weather stations along Taita hills transects; (A) *C*. *partellus* and *C*. *flavipes;* (B) *B*. *fusca* and *C*. *sesamiae* at Kipusi (832 m.a.s.l); (C) *C*. *partellus* and *C*. *flavipes*; (D) *B*. *fusca* and *C*. *sesamiae* at Vuria (1800 m.a.s.l).

In both low and high altitudes of the two transects the temperature is predicted to increase by 1–1.9°C throughout the year due to the climate change (Fig 7). This will lead to an increase in the finite rate of increase of *C*. *partellus* and *B*. *fusca* by 0.01–0.02 depending on the time of the year, and by 0.05 for both *C*. *flavipes* and *C*. *sesamiae* (Figs 8A, 8B, 8C, 8D,9A, 9B, 9C and 9D) However in both transects (Figs 8A and 9A) the change is more constant throughout the year at low than at high altitudes where the prediction suggests a weak change during the long dry season between May-September (Figs 8C and 9C); the prediction suggests no change of *C*. *partellus* activity at the tasseling stage of the maize. In the Kilimanjaro transect at lower altitudes, the change is more pronounced during the colder season (May-September) while almost no change is predicted during the warmer season (November-February) of *B*. *fusca* (Fig 8B). In the Taita Hills the changes are almost constant throughout the year along the entire transect, and there is a small increase during the colder season (July-September), when maize is grown during the long rainy season in lower altitudes, and during the long dry season in high altitudes (Fig 9A and 9C). Except at low altitudes in the Taita Hills, where *B*. *fusca* activity is predicted to increase at the beginning of the maize cycle, the predictions suggest very small changes in *B*. *fusca* activity at the beginning of the maize cycle in other localities when the maize is more vulnerable to stem borer attacks.

### Synchrony between the host borers and their respective parasitoids

The difference of ERI between *C*. *partellus* and *C*. *flavipes* (i.e. synchrony) (Figs 2K and 4K) ranges from -0.02 to 0.03 and -0.03 to 0.08 and between *B*. *fusca* and *C*. *sesamiae* (Figs 3K and 5K) and from -0.04 to 0.02 and -0.23 to 0.09 in Kilimanjaro and Taita Hills transects, respectively. These results suggest a disruption between the two pests and their respective parasitoid, which is more pronounced in Taita hills than in the Kilimanjaro transect, with a general trend indicating that in the future the two pests will be under better control by the parasitoids at low than high altitude.

### Predicting future maize losses

The proposed linear equation was found satisfactory for estimating the relationship between maize yield losses and the activity index of *C*. *partellus* (Table 1) and of *B*. *fusca* (Table 2). Under current temperature conditions, estimated average maize yield losses caused by *C*. *partellus* range from less than 2% in highland tropics to 23% on lowland tropics; the trend and range of variations are similar along both transects (Table 3). By 2055, the losses will increase by 5–7% along both transects in the four maize agro-ecological zones, but with a slightly greater increase in the Taita hills (Table 3). Under current temperature conditions estimated average maize yield losses caused by *B*. *fusca* range from less than 1% in the lowland tropics to more than 13% in the highland areas. Like for *C*. *partellus* the trend and range of variations are similar along both transects (Table 4). By 2055, the losses will increase by 13% in low altitudes (lowland tropic and dry mid-altitude) and by 21–23% in high altitudes (moist mid-altitude and highland tropics). The predicted increase in average yield loss due to *B*. *fusca* is much higher in the Taita Hills than in Kilimanjaro, i.e. almost 2 times in lower altitudes and between 4.3 and 17.1 times in higher altitudes (Table 4). If we consider both species together, the average increase in losses range between 7% in the highland tropics and more than 13% in the lowland tropics in the Kilimanjaro transect, and between 20% in the lowland tropics and almost 30% in the highland tropics in the Taita Hills. By 2055, the estimated increase in losses will overall be 2.5 times higher in Taita hills than in the Kilimanjaro region, as a result of the predicted temperature increase.

**Table 1:** Parameters of regression equations fitted to estimate the relationship between maize yield loss and activity index of *C*. *partellus*.

Transects	Intercept (*β*_0_)	Slope(*β*_1_)	df	F-stat	t-stat	P	*R* ^2^
Mount Kilimanjaro	-20.893(8.051)*	3.087(0.753)*	1	16.798	4.099	0.055	0.894
Taita hills	-27.961(10.998)*	3.844(1.059)*	1	13.155	3.627	0.068	0.868

*Figures in parentheses are standard errors.

**Table 2:** Parameters of regression equations fitted to estimate the relationship between maize yield loss and activity index of *B*. *fusca*.

Transects	Intercept (*β*_0_)	Slope(*β*_1_)	df	F-stat	t-stat	P	*R* ^2^
Mount Kilimanjaro	-39.554(16.468)*	6.855(2.370)*	1	8.364	2.892	0.102	0.807
Taita hills	-71.386(27.876)*	12.272(4.311)*	1	8.104	2.847	0.104	0.802

*Figures in parentheses are standard errors.

**Table 3:** Predicted losses in maize yield in Mount Kilimanjaro and Taita hills transects due to *C*. *partellus* infestation at current and future climatic conditions based on relationship between estimated yield loss and activity index.

			Mount Kilimanjaro			Taita hills		
			Predicted yield loss (%)			Predicted yield loss (%)		
ACZ	Altitude (masl)	Observed yield loss (%)*	Current (2013)	Future (2055)	Difference 2055–2013	Current (2013)	Future (2055)	Difference 2055–2013
**Highland tropics**	**>1600**	**0.01**	**2.84**	**8.34**	**5.50**	**1.74**	**7.74**	**6.00**
**Moist transitional**	**1300–1600**	**11.1**	**6.86**	**12.41**	**5.55**	**7.04**	**13.26**	**6.22**
**Dry mid altitude**	**1000–1300**	**11.09**	**12.14**	**17.85**	**5.71**	**14.66**	**21.19**	**6.53**
**Lowland tropical**	**<1000**	**22.69**	**23.06**	**28.10**	**5.04**	**21.42**	**28.11**	**6.69**
**Average**		**11.22**	**11.23**	**16.68**	**5.45**	**11.22**	**17.58**	**6.36**

ACZ are the agro-climatic zones, m.a.s.l. = metres above sea level.

* Estimated maize yield loss from Ong’amo et al. (2006).

**Table 4:** Predicted losses in maize yield in Mount Kilimanjaro and Taita hills transects due to *B*. *fusca* infestation at current and future climatic conditions based on relationship between estimated yield loss and activity index.

			Mount Kilimanjaro			Taita hills		
			Predicted yield loss (%)			Predicted yield loss (%)		
ACZ	Altitude (masl)	Observed yield loss (%)*	Current (2013)	Future (2055)	Difference 2055–2013	Current (2013)	Future (2055)	Difference 2055–2013
**Highland tropics**	**>1600**	**10.70**	**13.51**	**14.88**	**1.37**	**13.40**	**36.85**	**23.45**
**Moist transitional**	**1300–1600**	**14.60**	**10.97**	**15.77**	**4.80**	**10.77**	**31.67**	**20.90**
**Dry mid altitude**	**1000–1300**	**5.54**	**5.69**	**12.27**	**6.58**	**6.91**	**20.65**	**13.74**
**Lowland tropical**	**<1000**	**0.48**	**1.17**	**9.46**	**8.29**	**0.53**	**13.78**	**13.25**
**Average**		**7.83**	**7.84**	**13.10**	**5.26**	**7.90**	**25.74**	**17.84**

ACZ are the agro-climatic zones, m.a.s.l. = metres above sea level.

* Estimated maize yield loss from Ong’amo et al. (2006).

## Discussion

The predictive mapping of future maize stem borer pest risks at small scale generated through point-by-point analysis, using climatic data obtained from data logger dispatched along the two altitudinal gradients, confirm and complete results recorded at country scale generated from temperature extrapolated from available historical records by Khadioli *et al*., [51]. It confirms the precision of the temperature process based phenology model achieved with ILCYM software compared to other methods like rule based methods [62] and multivariate statistical techniques [63]. The results indicate a worsening of the impact of the two stem borer pests on maize production along the two East African mountain gradients studied. The aggravation can be attributed to three main changes occurring simultaneously: range expansion in higher altitude areas; increase of the abundance, thus damage potential, of the pests at all altitudes; disruption of the biological control due to a mismatch of the geographical distribution between the pests and their main larval parasitoids.

In both transects, *C*. *partellus* is the dominant species in areas belonging mostly to dry mid-altitude and dry transitional maize agro-climatic zones below 1200 m.a.s.l, reported as a zone with a low yield potential [53] while *B*. *fusca* is the dominant species in areas belonging mostly to moist mid-altitude and highlands tropic maize agro-climatic zones above 1200 m.a.s.l. reported as zones with a high yield potential [53]. The predicted increase in climatic suitability for establishment and survival will allow range expansion of both pests in altitudes above 1200 m.a.s.l. and suggests a future increase in the proportion of *C*. *partellus* in moist mid-altitude and moist transitional areas in both gradients. Our results are consistent with expansion of *C*. *partellus* to cooler zones reported in the last two decades in South Africa [45,46,47,48], and Ethiopia [64]. Similar range expansion to high altitude areas in East Africa have been reported recently for the coffee berry borer (*Hypothenemus hampei*) [65] and of the fruit fly *Bactrocera invadens* [66]. As for stem borers, several factors related to the competitive superiority of *C*. *partellus* against *B*. *fusca* were put forward to explain its expansion, among them a shorter generation time [42] and faster termination of diapause [42,45], but none of these studies pointed out the potential influence of the temperature change. However, over the past 40 years, Kenya’s average annual temperatures increased by 1°C with an increase of 0.5°C in western Kenya and 1.5°C in the drier parts in the East of Kenya [67]. The present findings suggest that temperature increase was a key factor responsible for the expansion of *C*. *partellus* to higher altitudes during the past 50 years.

According to our predictions of the change in abundance and finite rate of increase of both stem borers, which are based on life history traits generated in laboratory experiments [51,52], stem borer densities will increase in all maize agro-climatic zones along the two altitudinal gradients. More importantly, by 2055, maize agro-climatic zones above 1200m.a.s.l. such as the moist mid-altitude, moist transitional and highland tropics considered as unfavourable for *C*. *partellus*, and the highland tropics, which are not very favourable to *B*. *fusca*, will become more suitable.

Predicted change in the distribution of the two pests largely coincided with that of their parasitoid. However a slight disruption of the biological control is predicted with an improvement of the biological control of the two pests at altitudes below 1200 m.a.s.l. and a deterioration above 1200 m.a.s.l. This should lead to more frequent and severe stem borer outbreaks with potential aggravation of the already predicted aggravation of damages above 1200 m.a.s.l. in the most productive maize areas. The lower performance of *C*. *flavipes* in areas above 1200 m.a.s.l has already been reported by Zhou *et al*., [47] and was speculated to be due to lower occurrence of *C*. *partellus*. More recently, Mailafiya *et al*., [68] showed that the occurrence of both parasitoid species was influenced by geographic range of their respective host suggesting their distribution may be largely driven by the distribution of their old host association. Considering the predicted future changes in stem borer composition with a higher proportion of *C*. *partellus* in moist mid-altitude and moist transitional areas of both gradients, we could reasonably expect a distribution shift of *C*. *flavipes* to higher altitudes. However, the duration of the dry season has a strong influence on the availability of the two *Cotesia* species across seasons in Kenya [68]. Predicted climate change indicates that large parts of Kenya will experienced a 100 millimeter (mm) decline in the long-season rainfall by 2025 [69] with an increased frequency of dry years suggesting *Cotesia* spp. activities will be significantly affected in the future.

Based on the relationship between activity index of both stem borer pests and estimated maize yield losses in Kenya from De Groote [61] and Ong’amo *et al*., [48], maize losses will significantly increase in all agro-ecological zones where maize is grown along both transects but much more in lower areas of Kilimanjaro and across the entire Taita Hills transects. However, these predictions based on pests only do not consider the influence of temperature increase on the natural enemies. Our predictions show a small decoupling between both stem borer pests and their respective larval parasitoids suggesting higher pest numbers and more serious outbreaks than predicted with stem borers only. In addition, they do not take into account yield responses due to the climate change (changing rainfall amounts and patterns, temperature increase). Predictions made by Thornton *et al*., [70] suggest that by 2050, maize yields in East Africa will increase in highland areas of many parts while it will decrease in most lower elevations.

The predicted distribution trends for both stem borers are very similar for both transects. However *B*. *fusca* is recorded at all altitudes in the Taita Hills while in the Kilimanjaro transect it occurs at altitudes ranging from 1100–1600 m.a.s.l. The difference between the two transects suggests that other factors play a role in the establishment of stem borer pests. Our phenological model is purely based on temperature and does not take into consideration other climatic factors such as rainfall and relative humidity and, non climatic factors such as planting pattern and frequency, diapauses or other physiological factors that enable escape of harsh abiotic conditions. In addition, these models assumed no immigration and no emigration influencing population dynamics Thus, Sithole [71] argued that temperature, rainfall and relative humidity were the main factors affecting *C*. *partellus* distribution, with temperature being the most important. Abraham *et al*., [72], through correlation analyses, found that there was a combined influence of rainfall, relative humidity and mean minimum temperature. Due to the duration of the maize-cropping seasons ranging from 3 to 6 months depending on the altitude and the transect, the maize off-season never exceeds three months in Taita Hills whereas in Kilimanjaro it reaches almost nine months. Perennation of the stem borer pests during the dry season occurs mainly through diapause in crop residues and most likely depends also on the presence of suitable wild host plants such as wild sorghum (Sorghum arundinaceum L.), and guinea grass (*Megathyrsus maximus* (Jacq.) B.K.Simon & S.W.L.Jacobs) commonly found in the fallow surrounding maize plots. The duration of the diapause affects the survival rate of the larvae [73,74]. Thus predictions generated by the model should be interpreted with caution, always be coupled with field observations for a better understanding of the field dynamics of the pest.

In conclusion, altitudinal gradients are optimal spatial analogues, providing at small scale a range of different ecological conditions with variations of abiotic and biotic factors [18,75,76]. Our predictive mapping at small scale corroborates and completes results generated at country and even continental scale [51]. It confirms that temperature is a key factor explaining the distribution of insect pests and demonstrates the impact the predicted increase of temperature will have on the maize yields at all altitude but particularly in the most productive maize areas in the mid and high altitudes of the Eastern Afromontane Biodiversity Hotspot regions.

However, our results point out the likely role played by other climatic factors and by factors related to the top-down regulation of pests by parasitoids (host-parasitoid synchrony). During the past three decades, a general trend of decrease in rainfall was observed in East Africa during the long rainy season [69,77]. Despite uncertainty of the projections [4], if confirms, the future drought spells will become more frequent. In view of the high susceptibility of maize to droughts, the projected changes will most likeky exarcerbate maize yield reductions. Therefore, drought-tolerant maize varieties could play an important role in the adaptation to climate change in the next decades [78]. Other management changes of maize cropping systems like date of planting and cultural association should also be considered [16]. In addition, compared to maize, sorghum, pear millet or cassava are better adapted to higher temperatures and sporadic rainfall; they could be alternative crops to maize as an adaptation response to climate change in lowland areas [70,79,80,81].
